# Supplementary material for: A multi-dimensional analysis of CBEC English genre variation in South Asia: Based on Daraz
Source: PLoS One. 2023 Apr 13;18(4):e0279716. doi: 10.1371/journal.pone.0279716 (PMC10101417; doi:10.1371/journal.pone.0279716)
Supplement: Attachment — Submitted filename: Response to Reviewers.docx [file pone.0279716.s002.docx]

Dear editor and reviewers,

Thank you for reviewing my manuscript and sending me the letter for revised version.

To reply to questions raised by reviewer 1, I have added more explanation on why Crown and CLOB is considered as common language corpus and the reference corpus.

CROWN/CLOB corpus of contemporary English is modeled after the sampling strategies of Brown family corpora and established by Chinese scholars. CROWN/CLOB corpus matches its Brown family predecessors (such as Brown, LOB) in size and composition on the other and has been viewed as a good reference corpus for contrastive research of various kinds. The sources of publication come from U.S.-based texts and U.K.-based texts which were written by the U.S. and the U.K. citizens or permanent residents are selected. In the case of multiple authors, the primary/first author should hold U.S. or U.K. citizenship or permanent residence. Hence, the sources of selected texts could provide extremely native and precise British English and American English for academic research.

Due to historical reasons, British English as the first foreign language and even official language has been already imposing an essential influence on people’s daily life in South Asia. Besides, with entry into fast development era of internet and e-commerce, U.S. e-commerce platforms and social media, like Amazon, Twitter, LinkedIn, have been permeating most levels of South Asian society, along with American English.

To reply to questions raised by reviewer 2, I have made necessary corrections. Meanwhile, all the references mentioned in this paper are listed after the main body.

Moreover, I finish required revision on 9 aspects for more clarity.

1.Abstract: it has been corrected to be simple and precise enough to describe the

study purpose, methods and the findings. And the acronym were changed into fully-spelled words.

2. Literature review:

(1) MDA: I have added more elaboration around six dimensions. However, there are limited literatures around MDA and its essential tool, MAT, a statistical software. In initial submission, most of the literatures I could try my best to search abound MDA were all adopted in this paper. Due to its complexity of statistical approach and metadata privacy, MDA has not been widely applied in linguistic study since 1988. Until Multidimensional Analysis Tagger (v. 1.3) was developed by Nini in 2015, some scholars began to apply MDA to research.

3. I added elaboration with references around reference corpus Crown and CLOB. While observed corpus, including sub-corpora in this research, is a new self-constructed corpus. I added some brief introduction of sub-corpora in specific details to section 3.2.

4. I rearranged the order of 4 research questions with the logic of “what” first, followed by “why”: 1) What are the different genre features of CBEC English in South Asia? 2) Is there a genre difference among the four sub-corpora? 3) what are the main linguistic features in the corresponding dimensions that generate the genre differences? 4) What are the environmental factors for the occurrence of genre variation?

5. After reviewing the literatures again, the cohesion has been strengthened.

6. With the logic of research questions and the principle of context consistency, I rearranged the paper structure, especially the structure of Section 4 and added a sub-title 4.4.

7. I deleted new discussion in Conclusion section.

8. I have to admit the paper sounds a little lengthy due to the further discussion on more qualitative elaboration. However, I believe it is just the combination of genre differences along with linguistic and environmental factors that makes it an innovative and comprehensive research. After all, there has been a criticism aimed at the phenomenon of statistics complicated but linguistics naïve in corpus linguistics. Perhaps, I would delete some sentences to make it shorter and more concise.

9. I have revised spelling mistakes, sentence construction and grammar errors accordingly.

For Journal Requirements:

1. Manuscript has been corrected to meet PLOS ONE's style requirements, including those for file naming.

2. For my minimal data set, I just provide the value set of 67 linguistic features or variables in observed corpus. Further statistics analysis could be partly conducted based on the set.

3. I arranged Fig1 and 2 as required format both in the text and Figure files separately.

4. I reordered the Tables accordingly to make sure all of them referred in the text.

5. I added three references, No.28, No.38 and No.40, in the list to illustrate precisely the origin and application of Crown and CLOB. And I added No.46 that was missed in the initial manuscript.

I would like to thank you again for your valuable and significant comments.

I look forward to hearing from you regarding my revision. I would be glad to respond to any further questions and comments that you may have.

Yours

Shuang Wang
